# The key players of dysbiosis in Noma disease; A systematic review of etiological studies

**DOI:** 10.3389/froh.2023.1095858

**Published:** 2023-03-03

**Authors:** Ifeanyi Uzochukwu, David Moyes, Gordon Proctor, Mark Ide

**Affiliations:** Centre for Host-Microbiome Interactions, Faculty of Dentistry, Oral & Craniofacial Sciences, King’s College London, London, United Kingdom

**Keywords:** oral health, Noma, etiology, microbiome, microbiology, molecular biology, dysbiosis

## Abstract

Noma is a rapidly progressing periodontal disease with up to 90% mortality in developing countries. Poor, immunocompromised and severely malnourished children (2 to 6 years old) are mostly affected by Noma. Prevention and effective management of Noma is hindered by the lack of sufficient cohesive studies on the microbial etiology of the disease. Research efforts have not provided a comprehensive unified story of the disease. Bridging the gap between existing studies gives an insight on the disease pathogenesis. This current systematic review of etiological studies focuses on the key players of dysbiosis in Noma disease. This review was performed in accordance with the Preferred Reporting Items for Systemic review and Meta-Analyses (PRISMA) statement. Web of Science, MEDLINE *via* PubMed, Cochrane Library, Scopus, and Science Direct were searched electronically for clinical trials which applied culture dependent or molecular techniques to identify oral microbiota from Noma patients. Trials which involved periodontal diseases except Noma were excluded. After screening 275 articles, 153 full-texts articles were assessed for eligibility of which eight full text articles were selected for data extraction and analysis. The results show that 308 samples from 169 Noma participants (6 months to 15 years old) have been used in clinical trials. There was some variance in the microbiome identified due to the use of 3 different types of samples (crevicular fluid, subgingival plaque, and swabbed pus) and the ambiguity of the stage or advancement of Noma in the studies. Other limitations of the studies included in this review were: the absence of age-matched controls in some studies; the constraints of colony morphology as a tool in distinguishing between virulent fusobacterium genus at the species level; the difficulty in culturing spirochaetes in the laboratory; the choice of primers in DNA amplification; and the selection of probe sets in gene sequencing. This systematic review highlights spirochaetes and P. intermedia as putative trigger organisms in Noma dysbiosis, shows that F. nucleatum promotes biofilms formation in late stages of the disease and suggests that future studies should be longitudinal, with high throughput genome sequencing techniques used with gingival plaque samples from early stages of Noma.

## 1. Introduction

Noma is a ravaging orofacial gangrenous stomatitis which is characterized by acute necrotizing ulcerative lesions (1, 2). Noma is prevalent in developing countries where most of the victims are children (3–5). Epidemiological case study reports have established risk factors for the disease such as poor hygiene and nutritional status, measles and other eruptive fevers and immunocompromising diseases (6, 7). Although there are studies on Noma dated more than a century ago, the etiological organisms as well as the trigger agents are yet to be sufficiently detailed (8).

For decades, periodontal diseases were reported as infectious diseases caused by singular organisms, but more recent studies have established the host’s response to microbial dysbiosis as pivotal in the pathogenesis (9, 10). Dysbiosis is a microbial community shift or loss of homeostasis which is detrimental to human health (11, 12). Such detrimental effect is influenced by an alteration in ecological diversity, decrease in beneficial species, and an expansion of pathobionts (13, 14). Periodontal diseases are driven by complex dysbiosis of the oral microbiota (15, Deng et al. 2017b). Key players in the microbial community perform a transitory role from healthy state to dysbiosis (16, 17, Wang et al*.* 2012). Prevalence of these key players inflame the periodontal conditions such that commensal microorganisms are unable to thrive (18, 19). Although some research findings show that the products of metabolic activity in commensal bacteria play a role in periodontal diseases, the initiation of dysbiosis in Noma has not been thoroughly explored (20, 21).

The application of microbial and molecular methods in microbial ecology has elucidated the potential causative agents or trigger organisms in the pathogenesis of several diseases (22, 23). These breakthroughs have resulted in the prevention, diagnosis, and treatment interventions of diseases (24–27). This review of all primary etiological studies on Noma was carried out to establish the extent of consistency in the determination of the oral microbiome and key players of dysbiosis related to Noma disease, to highlight the constraints of the research studies, and to recommend improved strategies for future etiological studies.

## 2. Methods

This review was performed observing the Preferred Reporting Items for Systemic review and Meta-Analyses (PRISMA) statement (28). A search was conducted electronically in the following database for related papers: Web of Science; MEDLINE *via* PubMed; Cochrane Library; Scopus; Science Direct. This search used “Cancrum Oris” or “Noma” as context as well the following terms and their combinations: “Isolation” or “bacterial” or “clinical” or “microbiota”. Clinical trials which investigated the microbiome or characterized microorganisms from Noma patients either by culture dependent or molecular techniques were included. Clinical trials which characterized microorganisms from the oral cavity of patients with periodontal or gingival diseases which were not explicitly reported as Noma were excluded. Clinical trials with a generalized deficient description of microorganisms were excluded. Publications which were not available online were sourced directly from corresponding authors. The final search was performed on 29 September 2022.

Screening of the title and abstract was performed by the first and last author. The eligibility criteria used were: Primary study, clinical trials, Noma disease, and human patients. The following data items were extracted from searching the full text: article title, first author, year of publication, country of origin of sample population (Table 1). The analysis was conducted manually by reporting individual parameters of the included article in depth. These areas of interest were explored included the subject description, sampling details, methodical approach, method of identification, organisms identified & their relative prevalence, unique features of each clinical trial, bias & limitations of each experimental design.

**Table 1:** Included studies showing the title, year of publication and the country of origin.

Title	Year of publication	Country of Origin
Isolation of Fusobacterium Necrophorum from Cancrum Oris (Noma)	1999	Nigeria
Prevalent Bacterial Species and Novel Phylotypes in Advanced Noma Lesions	2002	Nigeria
Pro- versus anti-inflammatory cytokine profile in African children with acute oro-facial noma (cancrum oris, noma)	2005	Nigeria
Noma (Cancrum Oris) in Human Immunodeficiency Virus Infection and Acquired Immunodeficiency Syndrome (HIV and AIDS): Clinical Experience in Zimbabwe	2008	Zimbabwe
Bacterial Diversity in Oral Samples of Children in Niger with Acute Noma, Acute Necrotizing Gingivitis, and Healthy Controls	2012	Niger
Risk factors for noma disease: a 6-year, prospective, matched case-control study in Niger	2013	Niger
Microarray Analysis of Microbiota of Gingival Lesions in Noma Patients	2013	Niger
Noma Affected Children from Niger Have Distinct Oral Microbial Communities Based on High-Throughput Sequencing of 16S rRNA Gene Fragments	2014	Niger

## 3. Results

### 3.1. Included studies

**Figure 1:**
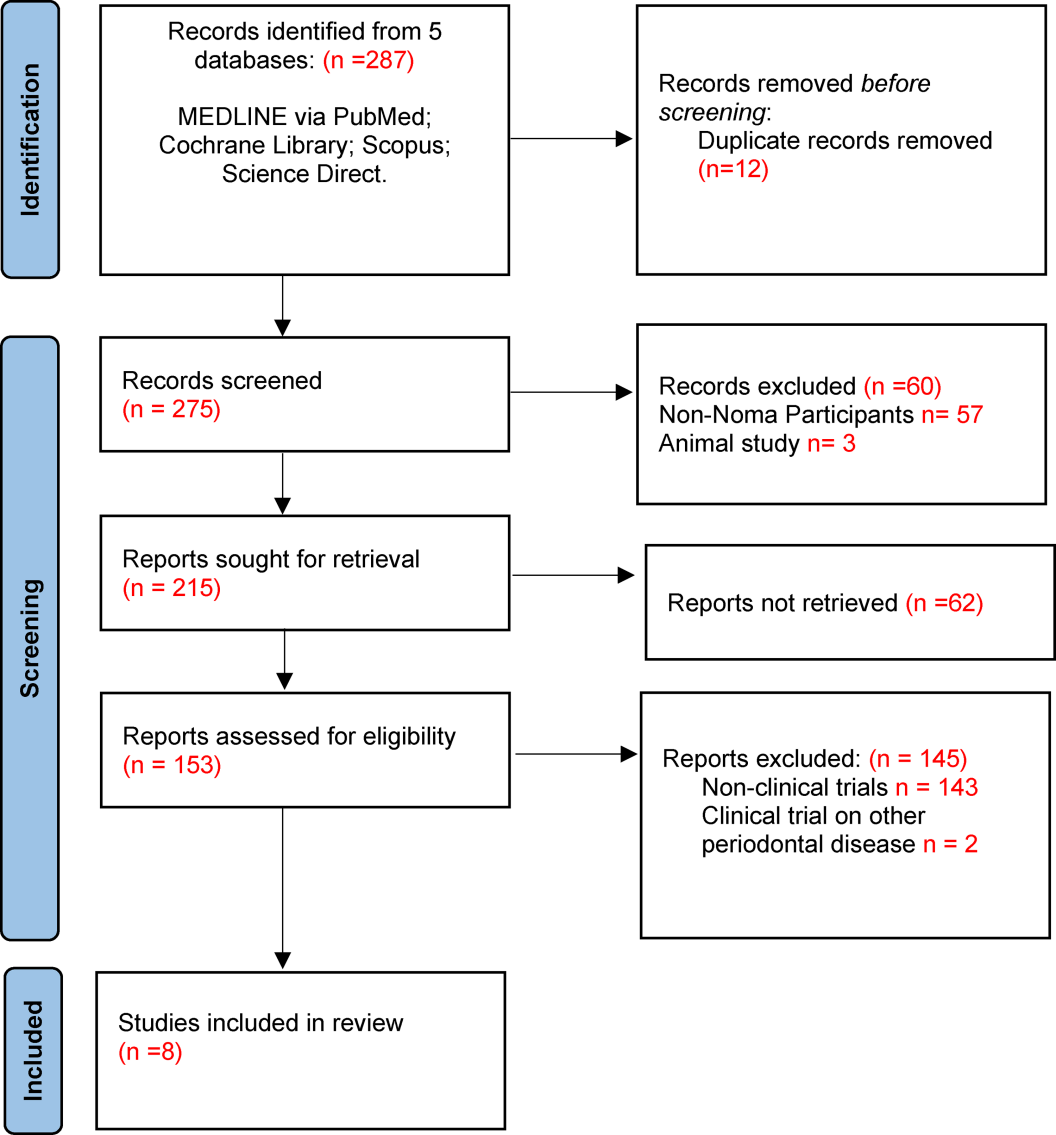
Process of screening literature.

The search was completed on 29 September 2022. A total of 287 article titles were retrieved from five databases. Upon removal of duplicates, 275 articles were then screened, and 153 full-texts articles were further assessed for eligibility. A total of 145 articles were excluded and 8 full text articles were included for data extraction and analysis (Figure 1). Considering the limited research publications about Noma disease, the studies included were of sufficient quality for this review.

The dates of the eight full text articles included in the data extraction spanned the period from 2009 to 2014. Although authors of the publications were globally distributed, the etiological studies were carried out on participants from the Western and Southern geographical regions of Africa namely, Nigeria, Niger, and Zimbabwe (Table 1). Further, all publications were identified as cross-sectional studies with case-control matching based on the Noma disease factor.

### 3.2. Sampling details

A total of 308 samples from 169 Noma participants and at least 747 case control participants have been used in etiological studies of Noma (Table 2). The age range of the participants span from 6 months to 15 years old. All Noma patients were assessed as malnourished based on standardized protocols of dietary history, anthropometric assessment, and plasma levels of nutrients. There was no categorization of the stage of Noma in the participants and only one study has reported other pre-existing immunocompromising conditions (HIV/AIDS). The samplings were performed at the dento-gingival sulcus at the active sites of lesions, damaged tissue, and the teeth at diseased dentition sites. Only 3 types of samples were used in the studies (highlighted in Table 2): crevicular/gingival fluid, subgingival tooth plaque, and pus swab. Clinical reports which described spirochetes and Fusobacteriales prior to these studies were excluded from the results presentation due to incomplete or inexplicit classification of isolates.

**Table 2:** Comparison of the Methodological approaches of etiological studies of Noma disease.

Study	Subject	Sampling details	Methodological approach	Identification
Falkler et al. 1999	3–15 years old	8 samples from active sites of Noma lesions:	Culture dependent: pre-reduced Brucella blood agar supplemented with hemin (0.05%) and menadione (0.1%) (BBHK)	Presumptive identification by colonial morphology, gram stain, phase contrast microscopy, and the air tolerance test.
	Malnourished; based on dietary history and anthropometric assessment			
		Gingival fluid from dento-gingival sulcus with the use of Sterile endodontic paper points		
			Pre-reduced selective fusobacteria agar Anaerobic incubation at 37°C for 4–5 days.	
				Anaerobic isolates identification
				using the AN-IDENT system
				Streptococcus isolates were biochemically identified using the API-20S
Paster et al. 2002	5 to 15 years old	4 samples from active sites of Noma lesions:	Culture independent: Amplification of 16S rRNA cistrons by PCR and purification of PCR products.	Species and closest relatives were identified by comparison of known species or phylotypes with 500 bases from 212 cloned inserts.
	Malnourished; based on dietary history and anthropometric assessment			
		Gingival fluid from dento-gingival sulcus with the use of Sterile endodontic paper points		
				Sequences of approximately 1,500 bases were obtained for most of the possibly novel species.
Phillips et al. 2005	Children (No age range provided)	6 crevicular fluid specimens collected by paper points for PCR.	Culture independent:	
			Undetailed Polymerase chain reaction methods were used to determine the presence of specific bacteria in oral samples	
	Malnourished; based on plasma levels of nutrients			
Chidzonga & Mahomva, 2008	2–5 years old	5 Pus swab samples from unspecified oral region	Undetailed microscopy and culture dependent studies	
	All patients were HIV-positive Malnourished; Low weight for age			
		Only 5 out of 48 patients had microbiological investigations conducted		
Bolivar et al. 2012	<12 years old	78 subgingival plaque samples of teeth in the premolar/deciduous molar region.	Extraction of total genomic DNA.	A total of 1237 partial 16 S rRNA sequences representing 339 bacterial species or phylotypes were obtained
	23 acute Noma cases			
			PCR amplification with broad specificity primers for the 16 S rRNA gene.	
	Age-matched control of the same sex.			
		Noma; diseased oral site (*n* = 23)	Seven libraries were generated from seven pools according to these category (noma, healthy, or acute necrotizing gingivitis), gender, and site status (diseased or control site).	
	9 Acute Necrotizing Gingivitis subjects			
				Sequences that were 99% to 100% similar to a fully named GenBank sequence were named likewise.
		Noma; healthy oral site (23)		
		controls (23)		
		Acute Necrotizing Gingivitis subjects (9)		
				The seven libraries were subjected to K-means cluster analysis.
Baratti-Mayer et al. 2013	<12 years old	117 microbial Samples subgingival plaque of teeth from Noma cases (59 from diseased dentition sites and 58 from healthy sites) and 235 from controls.	Extraction of total genomic DNA.	Scanned 132 sequences showing an abundance of at least 1% for probes a dataset containing against 1237 partial 16S rRNA gene sequences representing
	Malnourished.based on anthropometric indicators			
			Asymmetrical PCR with biotinylated universal primers.	
	All acute Noma cases.			
			Low-density 16S rRNA gene microarray.	
		62 eligible Noma cases	
				339 different phylotypes.
Huyghe et al. 2013	6 months -	84 Noma subjects, 37 ANG subjects, and 343 healthy controls	High-density and Low-density phylogenetic microarrays:	High-density arrays image analysis and signal quantification were achieved using Feature Extraction software
	12 years old			
	Nutritional status based on interviews and anthropometric indicator estimates			
			- Microarray design and manufacturing,	
		Gingival fluid from dento-gingival sulcus was collected from both lesion and non-lesion sites of each subject using sterile endodontic paper points.		
			- RNA/DNA extraction and quantification/biotinlyation PCR	
			- Microarrays hybridization and scanning	
				Low-density arrays signal intensities were extracted using IconoClust software Normalization and analysis were performed using the Partek Genomic Suite 6.4 (Partek, USA)
		Only one sample was taken from the mandibular anterior tooth of healthy controls		
Whiteson et al. 2014	6 months- 12 years old	60 Gingival fluid samples:	DNA extraction using the DNeasy Blood & Tissue Kit (Qiagen).	The V1-3 segment of the bacterial 16S rRNA gene was unidirectionally sequenced using 454 pyrosequencing technology.
	12 acute Noma cases	Noma healthy site (12)		
			Two-step PCR	
	12 age, location, and gender matched controls			
		Noma lesion site (12)		
			- Use of barcoded reverse primer in the amplification of the V1–3 region of the bacterial 16S rRNA gene	
	12 cases of Acute Necrotizing Gingivitis (ANG)			
		ANG healthy site (12)		
		ANG lesion site (12)	- Use of barcoded forward primer in the amplification of archaeal 16S rRNA gene. Aliquot from the first reaction used as template	
				Upon sequence read processing, Indicator Species Analysis (ISA) was carried out using the labdsv package in R The ISA score is calculated based on the relative abundance and frequency of each species by group.
		Control (12)		
				Monte Carlo randomization procedures were then used to test the statistical significance of the highest indicator values.

### 3.3. Culture-dependent microbial studies

The methodical approaches used in investigating the etiology of Noma are in two categories: the general culture-based method and the more species defining culture-independent methods (Table 2). The use of only pre-reduced *Brucella* blood agar and pre-reduced selective *Fusobacteria* agar represented 2.6% of the samples assayed. Beta-hemolytic *Fusobacterium necrophorum* was reported in 7 out of 8 diseased dento-gingival sulci (Table 3) and is present on both BHI agar and the *Fusobacteria* selective medium. *Prevotella intermedia* was identified in 6 out of 8 samples. Moreover, another microbial study identified *Staphylococcus aureus, Klebsiella* species, group D *Streptococcus*, and group B hemolytic *Streptococcus* as the predominant organisms in pus swabs from 5 Noma patients.

**Table 3:** Comparison of the results of etiological studies of Noma disease.

Study	Organisms & Prevalence		Unique features
Falkler et al 1999	Fusobacterium necrophorum	Percentage	Penicillin and tetracycline resistance shown by of F. necrophorum and *P*. intermedia respectively.
		87.5	
	Prevotella intermedia	75	
	Alpha-Streptococcus	50	
	Actinomyces spp.	37.5	
	Peptostreptococcus micros	12.5	F. necrophorum was present on both the BBHK agar and the fusobacteria selective medium
		12.5	
	Veillonella parvula	12.5	
	Pseudomonas spp.	12.5	
	Staphylococcus aureus		
Paster et. al 2002	Stenotrophomonas maltophilia	No. of clones	Only 1 out of 67 bacterial species or phylotypes detected was observed in previous study
		31	
	Ochrobactrum anthropi	16	
	Achromobacter xylosoxidans	10	
			Only one specie was observed in more than two subjects with the study
		7	
	Brevundimonas diminuta	4	
	Afipia genomospecies 8	4	
	Staphylococcus aureus	2	Results obtained using the spirochete-selective primers indicate that 85% of the clones have spirochetal inserts
	Propionibacterium acnes	2	
	Staphylococcus epidermidis		
Phillips et. al 2005	Prevotella intermedia	Percentage	Exclusion criteria included therapy with steroids or antibiotics or traditional medications within the preceding 48 h
	Porphyromonas gingivalis	83	
		83	
	Campylobacter rectus	50	
	Treponema denticola	50	
	Eikenella corrodens	50	
			Two age-matched control groups
Chidzonga & Mahomva, 2008	Staphylococcus aureus	Percentage	All patients were HIV-positive
	Klebsiella species	60	
	Group D Streptococcus	60	
	Group B Streptococcus	40	
	Pseudomonas species	20	
		20	
Bolivar et. al 2012	Prevotella Intermedia	K-Means Cluster	11 phylotypes of Spirochaetaceae accounted for 15 occurrences (81% in diseased sites)
	Peptostreptococcus stomatis	10.33	
		8.00	
	Prevotella intermedia	4.00	
Baratti-Mayer et. al 2013	Prevotella genus	Multivariant	Three age matched controls per sample and exclusion of 20 cases who received antibiotics.
	Neisseria genus	Odds ratio (95% CI)	
	Capnocytophaga genus	2.53(1.07–5.98)	
	Fusobacterium genus	3·24 (1.10–9.55)	
			Odds ratio from a multivariate conditional logistic regression model (*n* = 291)
	Spirochaeta genus	3·69(1.48–9.17)	
		4·63(1·61–13.35)	
		7·77(2·12–28·42)	
			Increased risk of disease when the Fusobacterium genus was under-represented. No molecular confirmation of *F. necrophorum* as a potential trigger organism
Huyghe et. al 2013	More represented in lesion sites	Underrepresented in lesion sites	Exclusion criteria included
	Prevotella intermedia	Fusobacteriales	children who had received antibiotic therapy, received fortified food in the 3 previous months, and had lesions older than 4 weeks
	Peptostreptococcus spp.	Tannerella spp.,	
	Nocardioidaceae	Cetobacterium spp.,	
	*P*. melaninogenica	Rothia spp	
	Prevotella nigrescens	Cardiobacterium spp	
	Porphyromonas endodontalis	Alcaligenaceae	
		Porphyromonadaceae,	
	Spirochaetaceae		Fusobacterium sp. was underrepresented in the lesion sites of Noma samples.
Whiteson et. al 2014	Prevotella	Abundance	Taxa indicated are more abundant in Noma or both Noma and ANG samples compared to the control.
	Spirochaetes	32	
	Peptostreptococcus	6.2	
	Atopobium spp	2.39	
		0.08	
			Treponema amylovorum was reported as indicator species for Noma.

### 3.4. Cloning and 16s rRNA gene sequencing

The first culture independent study on Noma etiology involved a subset of the sample population previously used in the microbial method. In 4 samples of gingival fluid (diseased) only *Staphylococcus aureus* was recurrent in both methods of identification (Table 3) and *Stenotrophomonas maltophilia* though undetected through microbial culture, was identified in more than two of the four samples by sequencing. The second 16S rRNA sequencing of crevicular fluid from Noma patients showed divergent results. *Prevotella intermedia* and *Tannerella forsythia* were identified in 5 out of 6 samples.

### 3.5. 16s rRNA gene-based oligonucleotide microarray analysis

Low-density and high-density phylogenetic microarrays probed 92.5% of all samples in these reported Noma etiological studies. Although low-density 16S rDNA microarray analysis showed considerable independent associations between microbiota and Noma, it did not report a specific organism as the causative pathogen*.* While *P intermedia* was associated with Noma, *F necrophorum* showed no triggering association. *Fusobacterium nucleatum* complex was the main reported *Fusobacterium* species.

High-density phylogenetic microarrays also observed that the *Fusobacterium* genus was prevalent or more abundant in healthy controls than Noma lesions (Table 3). In fact, fusobacteriales such as *Streptobacillus moniliformis*, *Cetobacterium,* and *Leptotrichia* had higher abundance in healthy donors than in Noma lesions. *Prevotella intermedia* was the main reported Prevotellaceae genus associated with Noma samples.

### 3.6. Limitations and bias in study designs

The limitations and bias associated with the study designs in etiological Noma research are in three categories: The lack of conformity in the type and site for sample collection; the lack of a valid control population to increase statistical reliability of results; the lack of a molecular assessment and analysis that is cognizant of the extensive biodiversity of the oral microbiota (Table 4). Due to the difference in presentation of results, the study heterogeneity could not be quantitatively analyzed by Meta-analysis.

**Table 4:** Bias and Limitations of etiological studies of Noma disease.

Study	Bias & Limitations
Falkler et al. 1999	• compelling discussion involved unpublished data of age-matched controls • No Information provided on patient therapy with antibiotics or traditional medications prior to sampling. • Incubation time of 4–5 days
Paster et al. 2002	• All Samples were collected from gangrenous lesions rather than early Noma lesions • No information provided on the use of controls • No Information provided on patient therapy with antibiotics or traditional medications prior to sampling.
Phillips et al. 2005	• Unspecified age range • No detailed information on the clones of the species
Chidzonga & Mahomva, 2008	• Collection of Pus samples instead of dental fluid • Pus swabs were collected from unidentified site of Noma lesions
Bolivar et al. 2012	• Pooling of samples into libraries will eliminate individual variation from the results and may allow one unusual sample to bias K-means cluster analysis. • Experimental bias either at the DNA extraction or the PCR amplification step may have led to underrepresentation of Spirochetes. the PCR reverse primer required at least one error to match *T. denticola* and other examples of uncultivated oral *Treponema*
Baratti-Mayer et al. 2013	• Ten phylotypes of Fusobacterium genus recorded belong to the *F. nucleatum* complex instead of the previously reported *F necrophorum* complex, however no data was shown to support this. • Due to density and technical factors, the microarray analysis was dedicated only to bacteria and some Archaea but did not detect fungi or parasites.
Huyghe et al. 2013	• The high-density phylogenetic arrays and low-density microarray characterized only a few taxa of the global bacterial profiles of the tested populations • Only 78.3% of the sequences of the RDP (release 9.34) were included hence some 16SrDNA sequences could not be assessed which resulted in a noncomprehensive probe set.
Whiteson et al. 2014	• 21% of initial sequence reads were excluded from clustering and analysis due to unmatched reverse primer, barcode, or homopolymer stretches greater than 6 nucleotides. • Although *Sharpea* showed the highest indicator value of Noma lesions (0.9626), it’s percentage of reads as determined by OTUs from the 97% cutoff was unreported for all sample groups. • Amplification of only the V1-3 segment of the bacterial 16S rRNA gene

## 4. Discussion

Distinct ecological habitats of various parts of the oral cavity represent different microbiota and disease dynamics (29, 30). The area of the oral cavity where the sample is collected plays some roles in the microbiome identified (31, 32). Although gingival crevicular fluid (GCF) has been extensively studied for antibodies and immunological inflammatory biomarkers of periodontal diseases (33, 34), saliva, supragingival and subgingival plaque are unequivocally preferred in the microbial characterization of periodontal diseases. Most studies (excluding 35) in this review carried out sampling from GCF and pus which raises the question of whether the organisms characterized are trigger microbes or merely a generic proliferation of the gingival microbiota in response to Noma disease. The analysis of GCF in chronic periodontitis showed that the distinguishing microorganisms and metabolites in these samples between periodontitis patients and healthy individuals are biomarkers (36). Saliva and gingival plaque may provide more information on the microbiota of periodontal dysbiosis than gingival crevicular fluid (37, 38).

All studies in this review were performed before the stage categorization of Noma disease was standardized (WHO Regional Office for Africa, 2016). As a result, there is evident disparity in the stage(s) of Noma disease reported in the sampled patients and the organisms identified. The sample population in many of the reviewed studies were patients with advanced lesions. These advanced lesions can be classified as stage 3 (Gangrenous), stage 4 (scarring), and stage 5 (sequelae) of Noma disease. At these stages, Noma is comparable to acute necrotizing ulcerative gingivitis (ANUG) (39, 40, Huyghe et al. 2013b). The identification of a trigger organism in post-virulent stages of Noma is therefore challenging and complex.

Although colony morphology is useful in distinguishing potential pathogens from normal flora, additional molecular techniques are required for discrimination of similar virulent species of the same genus (41). *Fusobacterium necrophorum* which causes pharyngotonsillitis and peritonsillar abscess but has not previously been linked with periodontal disease was identified by the first morphological study in this review (42). However, the absence of age-matched control groups and exclusion criteria for patients in this study poses a risk of bias in its design.

On the other hand, molecular studies in this review identified *F. nucleatum*, a periodontal pathogen which is ubiquitous in the oral cavity, as the main species in the Fusobacterium genus associated with Noma (43). The severity of periodontal diseases increases with the prevalence of *F. nucleatum* since it facilitates the formation of dental plaque (44, Han, 2015b). *F. nucleatum* is not considered a trigger organism in periodontal dysbiosis, although it connects the initial and later bacterial colonizers such that when *F. nucleatum* is absent, the prevalence of late colonizers is reduced (45, 46). *F. nucleatum* and *F. necrophorum* are the most frequent species implicated in *Fusobacterium* species bacteremia but morphological classification in the absence of molecular techniques such as 16s rRNA gene sequencing may lead to the misidentification of one for the other (47, 48).

Except for *Fusobacterium* species, the microbiota observed in ANUG is analogous to that described by molecular studies in this review (49, 50). Although this supports the theory of Noma as a dysbiosis, it does not support *P. intermedia* as the etiological trigger pathogen due to the advanced stage of the disease in which this species was characterized in (Darveau, Tanner, and Page, 1997 51). Previous studies have established the role of *P. intermedia* in the initiation and development of periodontitis by promoting periodontal connective tissue and bone matrix destruction through upregulated matrix metalloprotease production (52, Socransky et al. 1998b). Therefore, longitudinal molecular studies on the progression of simple gingivitis and acute necrotizing gingivitis to Noma sequelae will give a clearer picture of both the dysbiosis and trigger microorganism of Noma.

Like *P. intermedia,* oral *spirochetes* have been linked to necrotizing ulcerative gingivitis and other periodontal diseases. Particularly, clinical research has shown a correlation between the prevalence of *Treponema denticola* in periodontal pockets and the progression of periodontal disease in patients (53). Likewise, the higher representation of spirochaetes in Noma lesions is suggestive of its possible role in the dysbiosis of Noma. However, this was only reported in the molecular studies (35, Huyghe et al*.* 2013c). Due to the difficult nature of culturing *Spirochaetaceae* in the laboratory, specific culture conditions and specialized media are essential to optimize their growth and isolation (54, 55). Studies have demonstrated that *Spirochaetaceae* growth is only noticeable 4 weeks after culture and reaches its maximum within 8–12 weeks (56, 57). This contrasts with the longest incubation time of 5 days reported in this review. Also, *Spirochaetaceae* showed optional growth in Barbour-Stonner-Kelly (BSK) medium with rabbit serum, BSK swine serum + 5 fluorouracil, Cystine Tellurite Blood (CTB) medium, and brain heart infusion broth (56, 57). This potentially accounts for the absence of spirochaetes in the Noma samples since the culture dependent studies used Brucella blood agar supplemented with hemin (0.05%) and menadione (0.1%) (BBHK) (58, Chidzonga and Mahomva, 2008b).

The choice of primers in DNA Amplification (PCR) influences the microbiota detected and quantified using molecular techniques in the studies examined here, especially at the phylum level (59). 16S rRNA gene sequencing which used universally conserved primers in PCR read did not show a high representation of *Spirochaetaceae.* Whereas using the same sample population, PCR which used spirochete selective primers indicated that 85% of the clones have spirochetal inserts. *Spirochaete* species have a guanine (G): cytosine (C) ratio ranging from 51% to 65% in DNA (60, 61). This phenomenon not only segments DNA into several linear pieces, but it also makes it a difficult target for universal primers in DNA-DNA Hybridization (62, 63). This further suggests that the amplification achieved by Real-Time qPCR might not be a true reflection of the triggering possibilities of *Spirochaete* species in the dysbiosis of Noma disease.

The last four studies in this review demonstrate the influence of different molecular techniques on the etiological findings of Noma studies. Although conducted by the Geneva Study Group on Noma (GESNOMA) using the same sample population, the individual study designs and the limitations of each identification approach were discriminating factors in the results. The first 16S rRNA gene sequencing (Bolivar et al. 2012) attributed the underrepresentation (K-mean) of *Spirochetes* in Noma lesions to possible experimental bias of PCR reverse primer. Nonetheless, 11 phylotypes of *Spirochaetaceae* accounted for 15 occurrences in diseased sites. The study also emphasized that it’s library cloning yielded ratios of species not absolute numbers. Therefore, an unexpected increase in the representation of non-related *Bacteroidetes* (83%), might have resulted in a matched decrease for some other related bacteria such as *Fusobacteriaceae* (44%).

The last study by GESNOMA amplified the V1-3 segment of the bacterial 16S rRNA gene prior to 454 pyrosequencing (44). V2-3 hypervariable regions are not suitable for distinguishing all bacteria at genus level. This is the only study that reported *Treponema amylovorum* as the *Spirochaetaceae* species for Noma instead of *Treponema Denticola.* High-throughput sequencing of the entire 16S gene more accurately discriminates bacterial species and copy variants (64, 65). The pyrosequencing analysis ranked *fusobacterium* as low Noma indicators while placing *Sharpea* as the highest indicator species of Noma. On the contrary*, Sharpea* are commonly associated with rumen samples from low-methane-emitting sheep (66). While the abundance of *Prevotella* was well described in the results, the exclusion of a significant 21% of initial sequence read might have biased the computational analysis.

## 5. Conclusion

Although this systematic review demonstrated that Noma is a periodontal dysbiosis with *Spirochaetes* and *P. intermedia* as putative trigger organisms, *F. nucleatum* also enhances the late colonization of biofilms in subgingival plaque of advancing lesions. However, the limitations and therefore the bias of the study designs in this review demands stronger evidence from further studies. Longitudinal studies on the acute necrotizing gingivitis and oedema stages of Noma in endemic communities with age-matched controls is needed in the future. Such studies should employ high throughput full genome sequencing techniques on gingival plaque and saliva samples with primers and probe sets that are comprehensive to all possible oral microbiota or the use of PCR-independent molecular deep sequencing techniques.

## Data Availability

The original contributions presented in the study are included in the article/supplementary material, further inquiries can be directed to the corresponding author/s.
